# Diabetes Prevention, Early Intervention, and Nondrug Therapy

**DOI:** 10.1155/2017/5184250

**Published:** 2017-12-06

**Authors:** Ruozhi Zhao, Amy Leung Hui, Xinghai Yao, Chi Zhang, Feixia Shen

**Affiliations:** ^1^Department of Internal Medicine, University of Manitoba, Winnipeg, MB, Canada R3E 3P4; ^2^Phoenix Pharmaceuticals Inc., Burlingame, CA 94010, USA; ^3^Ruian Center of Chinese-American Research Institute for Diabetic Complications, Third Affiliated Hospital of Wenzhou Medical University, Wenzhou 325200, China; ^4^Department of Endocrinology and Metabolism, The First Affiliated Hospital, Wenzhou Medical University, Wenzhou 325000, China

The number of people with diabetes has risen from 108 million in 1980 to 422 million in 2014; the global prevalence of diabetes among adults over 18 years of age has risen from 4.7% in 1980 to 8.5% in 2014. Diabetes represents significant public health issue with potentially great financial burden of the government, families, and individuals; research addressing the prevention, early intervention, and nondrug therapy is urgently needed. In this special issue, we selected multiple original articles and two reviews which are aiming to explore diabetes prevention, early intervention, and nondrug therapy from clinical and basic research aspects.

Patients with prediabetes are at high risk for diabetes and cardiovascular disease (CVD). No study has explored whether the intervention could revert prediabetes to normal glycemic status as the primary outcome. Y. Luo et al. applied Beijing Prediabetes Reversion Program (BPRP) and evaluated whether intensive lifestyle modification and/or pioglitazone could revert prediabetic state to normoglycemia and improve the risk factors for CVD as well. Between March 2007 and March 2011, 1945 participants were randomized. At baseline, the individuals were 53 ± 10 years old, with median body mass index (BMI) 26.0 kg/m^2^ (23.9 and 28.2 kg/m^2^) and glycosylated hemoglobin (HbA1c) 5.8% (5.6 and 6.1%). 85% of the participants were impaired glucose tolerance (IGT), and 15% were impaired fasting glucose (IFG). Parameters relevant to glucose, lipids, blood pressure, lifestyle, and other metabolic markers were similar between conventional and intensive lifestyle intervention group at baseline. BPRP was the first study to determine if lifestyle modification and/or pioglitazone could revert prediabetic state to normoglycemia in Chinese population. Major baseline parameters were balanced between two lifestyle intervention groups. Z. Li et al. investigated the knowledge-attitude-practice (KAP) score in diabetes patients living in urban China regarding medical nutrition therapy (MNT) and explored the influencing factors. This national survey recruited diabetes and prediabetes patients in 40 hospitals across 26 provinces in China. A total of 6441 diabetes patients (mean age: 60.02 ± 13.14 years) completed this survey. The mean HbA1c level was 8.12 ± 2.12%, and the control rate of HbA1c (HbA1c < 7.0%) was 38.92%. Of the total, 53.56% had received MNT education. Over half of the patients had a poor total KAP score as well as poor K-, A-, and P scores. Patients with higher KAP scores had higher control rate of HbA1c (*P* < 0.05) but lower levels of fasting blood glucose (FpG) and 2-hour postprandial blood glucose (2hPG). Gender, occupation, residence, education level, and MNT education could influence the KAP scores (*P* < 0.05). This study showed that diabetes patients in urban China had poor understandings and practices related to MNT. Patients with higher KAP scores exhibited better control of blood glucose. J. Wang et al. assessed the effects of leisure-time physical activity on undetected prediabetes. A total of 8204 subjects were eligible for their analyses. For all subjects, high level of total leisure-time physical activity (OR = 0.78, 95% CI: 0.66–0.94) and low level of vigorous leisure-time physical activity (OR = 0.72, 95% CI: 0.58–0.90) were inversely associated with the risk of prediabetes in multivariate-adjusted model. For subjects under 45 years of age, high level of total leisure-time physical activity (OR = 0.78, 95% CI: 0.61–0.99) and low (OR = 0.61, 95% CI: 0.45–0.83) and high (OR = 0.72, 95% CI: 0.53–1.00) level of vigorous leisure-time physical activity were associated with a decreased risk of prediabetes. In the 45 to 65 age group, only high level of total leisure-time physical activity (OR = 0.73, 95% CI: 0.57–0.95) had a protective effect on prediabetes. Fulminant type 1 diabetes (FT1D) is considered to be an extremely rapidly progressing disease; previous literature reports that FT1D is a distinct subtype within type 1 diabetes (T1D) and most of the patients are adults. Y. Gu et al.'s multicenter study and cohort design involved sixteen hospitals; the total patient's number is 1470 from hospitalized newly onset T1D from Jan 2004 to Dec 2012. They found that FT1D onset age is much younger than that of classical T1D patients. The hospital-based incidence of FT1D in Chinese children was 1.56% in all new-onset T1D. In the aspects of treatment and prognosis, there was no significant difference between FT1D and classical T1D. Nailfold capillaroscopy is an easy and noninvasive technique used to investigate dermal microvasculature. S. Uyar et al. applied nailfold capillaroscopy and fundoscopic examinations in 216 patients with type 2 diabetes mellitus (T2DM) and 101 healthy control group, evaluated nailfold capillaries in T2DM patients, and determined the association of retinopathy with changes in the nailfold capillaries. They showed that retinopathy was detected 43.05% of diabetic patients (*n* = 93). In logistic regression analysis, tortuosity was shown significant (OR: 2.16; *p* = 0.036). There was also a significant relation between diabetes duration and most of the capillaroscopic findings. They suggest that capillarospcopic imaging could be a useful new technique for assessment of diabetic microvascular changes. Damage to small nerve fibers may develop in the early course of diabetes and can be assessed by sudomotor function testing. SUDOSCAN (Impeto, France) is a recently developed sudomotor function test of the electrochemical skin conductance (ESC) of the hands and feet, which has been used widely in early diagnosis of symmetrical diabetic neuropathy. SUDOSCAN can also be used for the efficient screening of cardiac autonomic neuropathy (CAN) using its proprietary cardiovascular autonomic neuropathy risk score (CAN-RS). D. Wang et al. reported that CAN-RS, a cardiac autonomic nerve dysfunction index calculated by SUDOSCAN, may be a promising index for the lens and vitreous abnormality screening in T2DM patients. T. He et al. demonstrated that ESC measurement is a reliable and feasible method to screen diabetic CAN in the Chinese population with diabetes before further diagnosis with cardiovascular autonomic reflex tests (CARTs). ANGPTL8 (angiopoietin-like protein 8) is a novel protein that primarily expressed in liver and fat. ANGPTL8 plays a role in regulating lipid metabolism in mice and in vitro tests. Y. Yin et al. reported that serum ANGPTL8 concentrations were significantly increased in IGR (impaired glucose regulation) and T2DM. Serum ANGPTL8 might play a role in the pathological mechanism of glucose intolerance. Patatin-like phospholipase domain-containing protein 3 (PNPLA3) polymorphisms serve as the genetic basis of hepatic steatosis (HS) in a normal population and lead to dysregulated glucose metabolism. Q. Pan et al. suggested that PNPLA3 rs1010023 may predispose chronic hepatitis B (CHB) patients with HS, but protects them from glucose dysregulation by attenuating the insulin resistance.

In a review article, Y. Zhao and H. Xing reviewed the relationship between diabetes mellitus (DM) and liver diseases from a relatively comprehensive perspective, including chronic hepatitis, cirrhosis, hepatocellular carcinoma (HCC), and liver transplantation (LT). The liver plays an important role in the regulation of glucose homeostasis. Viral liver diseases due to the presence of liver injury are risk factors of DM. Uncontrolled liver injury or glycemic level would accelerate the progression of DM, resulting in the survival of patients shortened. Treatment of diabetes may be difficult due to liver insufficiency and hepatotoxicity of antidiabetic drugs. Treatment of liver diseases would relatively improve the prognosis of the DM patients. Mesenchymal stem cells (MSCs), an ideal cell source for regenerative therapy with no ethical issues, play an important role in diabetic foot ulcer (DFU). Growing evidence has demonstrated that MSC transplantation can accelerate wound closure, ameliorate clinical parameters, and avoid amputation. Y. Cao et al. reviewed the mechanism of preclinical studies, as well as safety and efficacy of clinical trials in the treatment of DFU. Bone marrow-derived mesenchymal stem cells (BM-MSCs), compared with MSCs derived from other tissues, may be a suitable cell type that can provide easy, effective, and cost-efficient transplantation to treat DFU and protect patients from amputation.

The articles in this issue provide some new ideas for diabetes prevention, early intervention, and nondrug therapy. It is our hope that our readers will enjoy these articles and assist them in their future research.

